# Cervical Intradural Disc Herniation: Case Report and Operative Video

**DOI:** 10.7759/cureus.7537

**Published:** 2020-04-04

**Authors:** Sricharan Gopakumar, Visish M Srinivasan, Alexander Ropper, Michael Raber

**Affiliations:** 1 Neurological Surgery, Baylor College of Medicine, Houston, USA

**Keywords:** disc herniation, acdf, intradural, cervical

## Abstract

Cervical intradural disc herniation (CIDH) is a rare presentation of an intradural disc herniation. We present a case of CIDH with associated surgical video depicting an anterior surgical approach to treatment. Patient presentation, relevant radiographic imaging, surgical exposure and technique, and cerebrospinal fluid leak complication and prevention are discussed.

## Introduction

Cervical intradural disc herniation (CIDH) is exceedingly rare with less than 40 reported cases in the literature. We present such a case with an accompanying microsurgical video. Intradural disc herniations (IDHs) comprise less than 0.3% of all disc sequestrations, and cervical lesions comprise only 3% of all intradural herniation cases [1,2]. In contrast, lumbar and thoracic spine IDH cases comprise 92% and 5%, respectively [1]. The most common locations of CIDH found in systematic reviews include C5-6 (35.1%-43.5%), the level involved in our patient C6-7 (24.3%-30.4%), C4-5 (13%), and C3-4 (13%) [1,2]. Increased number of cases seen at the lower cervical spine is thought to be related to the high tension on the posterior longitudinal ligament (PLL) from neck movement at this level [2]. CIDH was spontaneous in 55.6%-61% cases, such as the case in our patient with no history of preceding cervical trauma [1,2]. Common presenting signs include Brown-Sequard syndrome, quadriparesis, and radiculopathy [1]. Our case and associated surgical video serve to illustrate an intraoperatively confirmed diagnosis of a CIDH.

## Case presentation

The patient is a diabetic, non-smoking 71-year-old Caucasian male with a history of hypertension and coronary artery disease who presented to the emergency room with acute-onset, rapidly progressive weakness and numbness in both his upper and lower extremities over the preceding day. The patient also describes a three-week history of bilateral shoulder pain that did not resolve with tramadol. He was able to ambulate the previous day but developed weakness in his lower extremities bilaterally. This soon progressed overnight to total loss of motor strength and lower extremity paraplegia. He also described bilateral upper extremity weakness, most notably in his hands where he had essentially no grip strength. There was no history of recent trauma.

On physical exam, motor strength was 5/5 in the deltoids bilaterally, 5/5 in the biceps bilaterally, 3/5 in the triceps bilaterally, 3/5 wrist flexion, 1/5 finger flexion, 0/5 hand intrinsics, and 0/5 in the lower extremities bilaterally with no lower extremity movement. A sensory level was noted at T6. The triceps, patellar, and ankle were areflexic with no clonus, and the patient had a negative Hoffman’s sign, no bulbocavernosus reflex, and no rectal tone.

**Figure 1:**
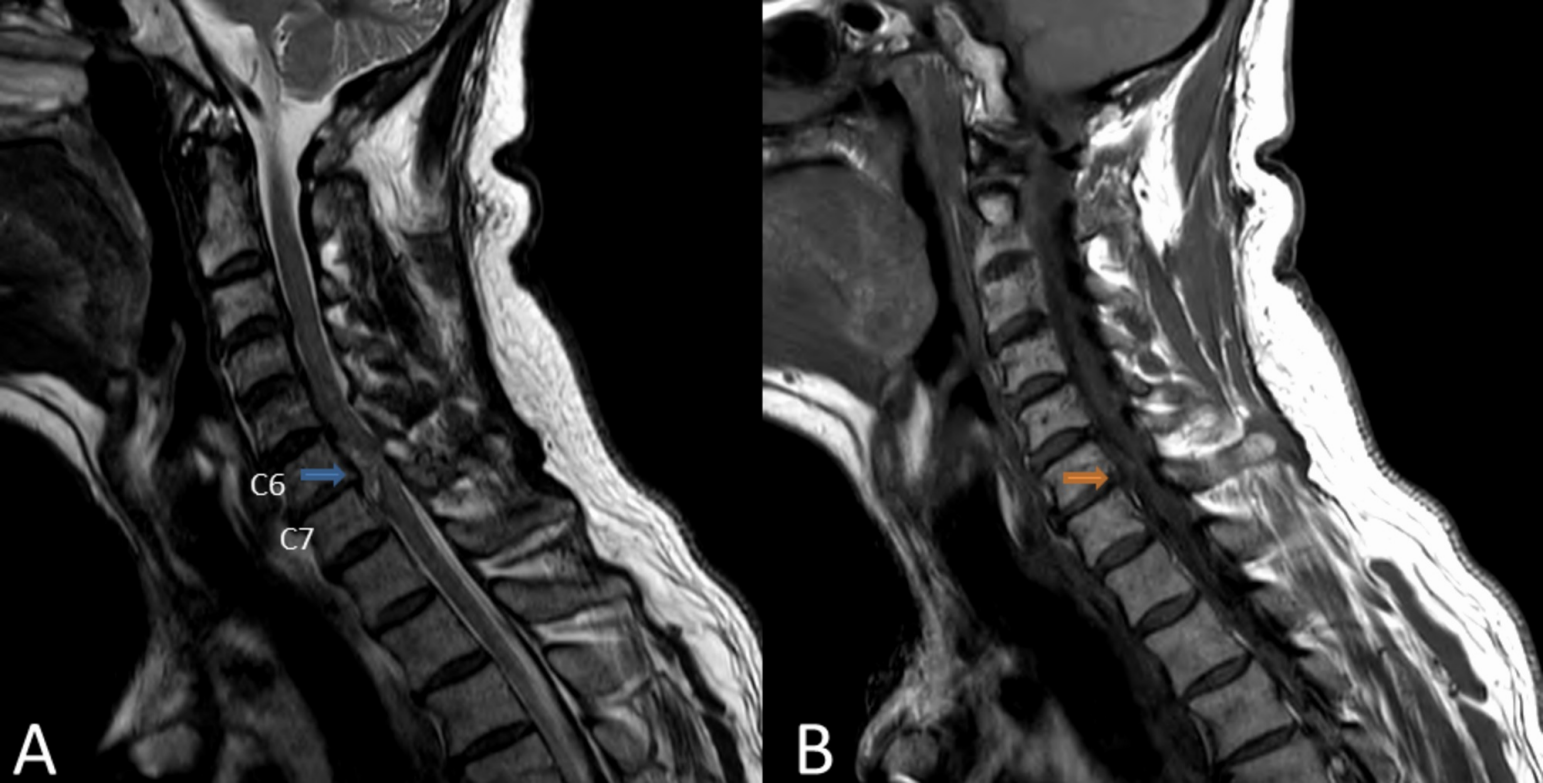
Sagittal MRI Sagittal T2 (A) and T1 (B) MRI demonstrated a cervical disc herniation at C6-C7 (arrows). The lesion was also read as a possible meningioma. The lesion extends behind each of the vertebral bodies rostrally and caudally. The spinal cord is severely compressed without evidence of myelomalacia.

MRI cervical spine was performed and revealed C6-7 ventral spinal cord compression with no evidence of cord contusion (Figure 1). The differential at this time included meningioma, hematoma, or abscess. The lesion was not definitively within the extradural or intradural space.

Preoperative dynamic x-rays were not performed, and the patient was taken for emergent surgical intervention as a spinal cord injury case. He was placed in standard anterior cervical discectomy and fusion (ACDF) positioning, and localizing was performed for a C6-7 ACDF. Care was taken to maintain appropriate spinal cord perfusion during transfers and induction of anesthesia, as well as gentle extension for the approach. A partial discectomy was completed. Upon initial opening of the PLL with a nerve hook, cerebrospinal fluid (CSF) egress was noted (Video 1). 

**Video 1:** Operative Video Operative video of the case, showing microdissection of the intradural disc herniation, leak repair, and completion of the ACDF. ACDF, anterior cervical discectomy and fusion.

Observation of CSF leak raised concern for a ventral dural defect. The dura was further exposed. There was no clear plane of dissection for the PLL from the dura. A nerve hook was used underneath the dura to sweep and separate the disc from the PLL and the dura. Upon further removal of the PLL, a midline, vertical slit dural defect with herniated disc material extending intradurally was discovered having a dumb-bell configuration with the small dural defect as the waist. The intradural portion of the disc was dissected circumferentially so the dural margins could be identified. The extradural component of the disc was truncated to facilitate further exploration of the dural defect. The dural hiatus was then gently retracted to expand the window and microdissection was performed circumferentially to loosen the disc herniation.

Eventually, the intradural disc was released and removed without any further direct expansion of the defect. The linear dural split was closed with fibrin glue dural sealant, as the narrow exposure did not allow for primary closure. Finally, an undersized graft was put in place to complete the ACDF, and a drain to gravity suction was inserted. Postoperatively, the patient experienced no acute complications. The patient’s neurological status was maintained, and the patient was discharged to a rehabilitation facility two weeks later.

## Discussion

Preoperative diagnosis of CIDH is challenging, with only 13% of cases diagnosed preoperatively and 87% of cases confirmed intraoperatively [1]. In approximately 5% of cases, a halo of CSF isointensity is present surrounding a herniated mass on sagittal T2 MRI, indicating intradural extension into the cervical spine [1]. Ossification or hypertrophy of the PLL can be appreciated on MRI or CT in a minority of cases [2]. The “Y-sign,” formed by the intradural herniation bifurcating the ventral dural line into the two separate lines of dura and arachnoid, has been reported as another indicator of CIDH on imaging [1].

During such intradural cases, it is important to have a controlled dural opening to the extent possible. Adhesion of the PLL and the ventral dura can be encountered and may play a role in pathogenesis of CIDH, which remains unclear [1,2]. CSF leak is a known complication of anterior cervical spine surgery that affects anywhere from 0.2% to 1% of ACDF or degenerative cervical spine cases [3]. Observing CSF leakage before dural incision indicates a breach of the arachnoid matter and requires repair using direct suture, fascia graft with fibrin glue, dural substitute, or fibrin glue alone, which was used in our case [1]. IDH cases in particular have increased risk of CSF leak that should be managed.

The durotomy can be managed according to a previously published protocol [3]. CSF leak can be addressed by first draining the CSF to lower pressure. Using cottonoid to dry the durotomy site promotes patch adherence and prevents dehiscence. Dural sealant, sometimes with a dural substitute, is used to indirectly repair the durotomy. An undersized interbody graft in the anterior posterior dimension is then used, with a smaller graft preventing sealant expansion into the spinal cord. Finally, a wound drain is placed to allow for passive drainage to gravity. Such management can prevent complications such as meningitis, headaches, wound breakdown, or the need for a lumbar drain or additional surgery [3]. Ultimately, surgical intervention can allow for a resolution of symptoms and carefully managed CSF leak is important for a positive patient outcome.

## Conclusions

CIDH is a rare presentation of IDH and spinal cord injury. It can be managed safely by a traditional anterior approach, but involves the additional challenge of managing a CSF leak. Careful microdissection is needed to safely remove the intradural fragment, compared to a typical disc herniation. However, the potential for direct injury to the spinal cord, including disruption of its sensitive microvasculature, may result in worse outcomes than typical cervical disc herniations.
